# miR-218-5p Induces Interleukin-1β and Endovascular Trophoblast Differentiation by Targeting the Transforming Growth Factor β-SMAD2 Pathway

**DOI:** 10.3389/fendo.2022.842587

**Published:** 2022-03-01

**Authors:** Yanan Shan, Yan Chen, Jelena Brkić, Leslie Fournier, Haiying Ma, Chun Peng

**Affiliations:** ^1^ Department of Biology, York University, Toronto, ON, Canada; ^2^ Centre for Research on Biomolecular Interactions, York University, Toronto, ON, Canada

**Keywords:** endovascular trophoblast, placenta, miR-218-5p, IL1β, TGFβ, SMAD

## Abstract

The acquisition of an endovascular trophoblast (enEVT) phenotype is essential for normal placental development and healthy pregnancy. MicroRNAs (miRNAs) are small noncoding RNAs that play critical roles in regulating gene expression. We have recently reported that miR-218-5p promotes enEVT differentiation and spiral artery remodeling in part by targeting transforming growth factor β2 (TGFβ2). We also identified *IL1B*, which encodes interleukin 1β (IL1β), as one of the most highly upregulated genes by miR-218-5p. In this study, we investigated how miR-218-5p regulates *IL1B* expression and IL1β secretion and the potential role of IL1β in enEVT differentiation. Using two cell lines derived from extravillous trophoblasts (EVTs), HTR-8/SVneo and Swan 71, we found that stable overexpression of miR-218-5p precursor, mir-218-1, or transient transfection of miR-218-5p mimic, significantly increased *IL1B* mRNA and IL1β protein levels in cells and conditioned media. We also showed that miR-218-5p directly interacted with SMAD2 3’UTR and reduced SMAD2 at mRNA and protein levels. Knockdown of SMAD2 induced *IL1B* expression and attenuated the inhibitory effect of TGFβ2 on *IL1B* expression. On the other hand, overexpression of SMAD2 reduced IL1β levels and blocked the stimulatory effects of miR-218-5p on *IL1B* expression, trophoblast migration and endothelial-like network formation. In addition, treatment of trophoblasts with IL1β induced the formation of endothelial-like networks and the expression of enEVT markers in a dose-dependent manner. These results suggest that miR-218-5p inhibits the TGFβ/SMAD2 pathway to induce IL1β and enEVT differentiation. Finally, low doses of IL1β also inhibited the expression of miR-218-5p, suggesting the existence of a negative feedback regulatory loop. Taken together, our findings suggest a novel interactive miR-218-5p/TGFβ/SMAD2/IL1β signaling nexus that regulates enEVT differentiation.

## Introduction

The placenta is a multifunctional transient organ essential for nutrient and gas exchange between the mother and the fetus throughout the pregnancy (1). During placental development, cytotrophoblast progenitor cells differentiate into two lineages, syncytiotrophoblasts and extravillous trophoblasts (EVTs). EVTs acquire invasive properties and further differentiate into interstitial trophoblasts and endovascular trophoblasts (enEVTs). enEVTs invade the uterus and replace the endothelial cells lining the maternal spiral arteries, and transform them into high flow, low resistance vessels. Insufficient enEVT differentiation, invasion, and spiral artery remodeling can decrease blood flow to the placenta and cause oxidative stress, which is known to precipitate the early onset (<34 weeks of gestation) preeclampsia (PE). PE is a major pregnancy-related disorder characterized by hypertension and multi-organ damage (2). It is a leading cause of maternal and neonatal morbidity and mortality and affects approximately 3%–5% of pregnancies worldwide (3).

The transforming growth factor β (TGFβ) superfamily plays a crucial role in the development and tissue homeostasis. Members of this family signal *via* heteromeric complexes of type I and type II receptors to activate receptor-regulated SMAD (R-SMAD), which form a complex with SMAD4 and translocate to the nucleus to regulate target gene transcription (4). Two R-SMADs, SMAD2 and SMAD3, are known to be activated by TGFβ1, 2, 3, Activin, and Nodal. These SMADs, together with the TGFβ ligands and receptors, are all expressed in trophoblasts (5–7). These signaling molecules regulate a variety of cellular functions, such as proliferation, migration, invasion, and apoptosis (8–13), as well as hormone production (14), and their dysregulation is associated with PE (15–18). Interestingly, we have recently found that SMAD2 and SMAD3 play differential roles in enEVT differentiation, in that activation of SMAD2 or inactivation of SMAD3 suppresses the acquisition of an enEVT-like phenotype (19).

MicroRNAs (miRNAs) are a class of small and highly conserved noncoding RNAs that are critically involved in numerous physiological and pathological events. In most cases, miRNAs interact with the 3′ untranslated region (3′UTR) of target mRNAs to induce their degradation and repress the translational process (20). The differential expression profiles of miRNAs in placentas from healthy and PE patients have been documented and some miRNAs have been reported to regulate trophoblast functions and placental development by modulating various signaling pathways, including the TGFβ pathway (21–24). For example, miR-195, downregulated in PE placental tissues, represses trophoblast invasion by targeting ACVR2A, a type II receptor for Activin and Nodal (25). miR-376c and miR-378-5 increase trophoblast proliferation, motility, and survival by inhibiting Activin receptor-like kinase (ALK) 5 (type I TGFβ receptor)/ALK7 (type I Nodal receptor) and Nodal, respectively, both leading to compromised TGFβ signaling (26, 27). In addition, we and others have found that the expression of miR-18a and miR-218-5p is decreased in placentas from PE patients (21, 28, 29). These two miRNAs stimulate EVT differentiation, invasion, and spiral artery remodeling through the inhibition of SMAD2 and TGFβ2, respectively (28, 29).

Interleukin 1β (IL1β) is a proinflammatory cytokine that may play a role in implantation (30). Several studies have reported that IL1β increases the invasive capacity of trophoblasts (31, 32) and enhances the secretion of IL8 from endometrial cells that subsequently stimulates survival and migration of first trimester villous cytotrophoblasts (33). However, IL1β may also have harmful effects on placental development, as serum IL1β levels are increased in gestational diseases, including PE and preterm labor (34–36), suggesting that a balanced IL1β expression/activity is important for a healthy pregnancy. To date, whether IL1β modulates enEVT differentiation has not been reported, and this merits investigation.

Recently, we have reported that miR-218-5p stimulates enEVT differentiation and spiral artery remodeling by inhibiting TGFβ2, and the *IL1B* mRNA is upregulated by miR-218-5p (28). In this study, we further investigated how miR-218-5p regulates IL1β and determined the potential role of IL1β in the acquisition of an enEVT-like phenotype. We hypothesized that miR-218-5p induces IL1β by targeting the TGFβ signaling pathway and that IL1β contributes to the miR-218-5p-induced enEVT differentiation. 

## Materials And Methods

### Cell Culture

HTR-8/SVneo (37) and Swan 71 (38) cell lines were developed from first trimester placentas. These cells resemble EVT in their invasive ability and the expression of EVT markers including HLA-G, cytokeratin 7, vimentin, ITGA1, and ITGA5 (19, 28, 38). Both HTR-8/SVneo and Swan 71 cells were obtained and cultured as previously described (19). Briefly, HTR-8/SVneo cells were cultured in RPMI 1640 medium containing L-glutamine (HyClone) and 10% FBS (GIBCO). Swan 71 cells were maintained in DMEM/F12 medium (HyClone) supplemented with 10% FBS. Cells were cultured at 37°C with 5% CO_2_ and were periodically checked for mycoplasma contamination using a mycoplasma detection kit (Biotool, Jupiter, FL, USA).

### Transfection and Recombinant Protein Treatment

HTR-8/SVneo cells stably overexpressing mir-218-1 were generated as previously described (28). Cells were seeded into 6-well plates and allowed to reach 70% confluence before transfection. Transient transfection of 100 nM miRNA mimics and 200 nM siRNAs (GenePharma, Shanghai, China; sequences are listed in **Table 1**) was conducted with Lipofectamine RNAiMax (Thermo Fisher Scientific, Burlington, ON, Canada). Transfection of 2 µg SMAD2 and/or SMAD3 expression plasmids (39, 40) was carried out using Lipofectamine 2000 (Thermo Fisher Scientific). All transfections were performed following the manufacturer’s protocols. After 6 hr, the medium was changed and cells were recovered in a growth medium for 24 hr, followed by total RNA isolation or functional assays. For protein expression, cell lysates were collected 48 hr after the transfection. In some experiments, cells were also treated with recombinant human IL1β (208-IL-010), TGFβ1 (243-B-002), TGFβ2 (302-B2-002), TGFβ3 (243-B3-002), or Activin A (338-AC-010) (all purchased from R&D Systems, Minneapolis, MN, USA) for 24 hr.

**Table 1:** Sequences of primers, siRNAs, and microRNAs.

Name	Sequence:
hsa-miR-218-5p mimic	Sense: 5’- UUGUGCUUGAUCUAACCAUGUtt -3’ Antisense: 3’- ttAACACGAACUAGAUUGGUACA -5’
Non-targeting Control (NC)	Sense: 5’- UUCUCCGAACGUGUCACGUtt -3’ Antisense: 3’ - ttAAGAGGCUUGCACAGUGCA -5’
siTGFB2	Sense: 5’- ACCAAATACTTTGCCAGAAACTATtt -3’ Antisense: 3’- ttTGGTTTATGAAACGGTCTTTGATA -5’
anti-hsa-miR-218-5p	RiboBio™ anti-hsa-miR-218-5p inhibitor
anti-NC	RiboBio™ miRNA inhibitor, negative control
miR-218-5p	F: 5’-TTGTGCTTGATCTAACCATGT-3’ R: N-Code universal primer
U6 snRNA	F: 5’-CGCAAGGATGACACGCAATT-3’ R: N-Code universal primer
TGFB2	F: 5’-ATTGATGGCACCTCCACATATA-3’ R: 5’-ACGTAGGCAGCAATTATCCTG-3’
CDH5	F: 5’-GCCAGTTCTTCCGAGTCACA-3’ R: 5’-TTTCCTGTGGGGGTTCCAGT-3’
ITGA1	F: 5’-GCTGGCTCCTCACTGTTGTT-3’ R: 5’-CACCTCTCCCAACTGGACAC-3’
ITGA5	F: 5’-ACATCTGTGTGCCTGACCTG-3’ R: 5’-CTGGAGAAGTTCCCTGGGTG-3’
PECAM1	F: 5’-ATTGCAGTGGTTATCATCGGAGTG-3’ R: 5’-CTCGTTGTTGGAGTTCAGAAGTGG-3’
SMAD2 3’UTR	F: 5’-AGGACTAGTATTCACAGGGAAGCTCATGG-3’ R: 5’-CCCAAGCTTATGGCGGTTTTGTCGAATAG-3’
SMAD7	F: 5’- CAGGCATTCCTCGGAAGTCA-3’ R: 5’- TGGACAGTCTGCAGTTGGTTT-3’
β-actin	F: 5’GACCTGTACGCCAACACAGT R: 5’-AGTACTTGCGCTCAGGAGGA

### Quantitative Real-Time PCR (qPCR)

Total RNA was extracted from cells using TRIzol Reagent (Thermo Fisher Scientific) according to the manufacturer’s protocol. RNA was reverse transcribed with Moloney murine leukemia virus (M-MuLV) reverse transcriptase (New England Biolabs, Whitby, ON, Canada). RNA purity and concentration were examined by a NanoDrop 2000 Spectrophotometer (Thermo Fisher Scientific). All the samples had high purity, as indicated by an A260/A280 ratio of 2.01~2.1 and an A260/230 ratio >2. The integrity of RNA was confirmed using agarose gel electrophoresis. miRNA reverse transcription was performed using a TaqMan^®^ microRNA reverse transcription kit (Thermo Fisher Scientific) with a unique reverse primer. qPCR was carried out using BlasTaq 2×qPCR master mix (Applied Biological Materials, Richmond, BC, Canada) and gene specific primers (**Table 1** **)**. miR-218-5p and the internal control were measured using the hsa-miR-218-5p TaqMan miRNA kit and U6 snRNA TaqMan^®^ control miRNA kit (both from Thermo Fisher Scientific), respectively. All qPCR assays were performed on Rotor-Gene Q (Qiagen, Toronto, ON, Canada). The relative mRNA and miRNA levels were calculated using the 2^-ΔΔCt^ method, normalized with β-actin and snRNA U6 as housekeeping control, respectively.

### Western Blotting

Cells lysates were collected by lysing cells with RIPA buffer (50 mM Tris/HCl, 150 mM NaCl, 1 mM EDTA, 1% Triton-X, 0.5% NP-40, 0.1% SDS, 1.0 mM DTT, pH 7.4) containing Pierce protease and phosphatase inhibitors (Thermo Fisher Scientific) on ice for 30 min. During the period, cell lysates were briefly vortexed every 10 min for 3 times, followed by centrifugation at 13000 rpm for 15 min at 4°C. Protein concentration was quantified using a Pierce™ BCA protein assay kit (Thermo Fisher Scientific). An equal amount of protein samples were separated by SDS-polyacrylamide gel electrophoresis and transferred to a PVDF membrane (Millipore Sigma, Burlington, MA, USA). The membrane was blocked with 5% blocking buffer (5% skim milk in TBST) for 1 hr and incubated with an anti-SMAD2/3 (3102S, Cell Signaling Technology, Danvers, MA, USA, 1:500) or anti-GAPDH (sc-365062, Santa Cruz Biotechnology, Dallas, TX, USA, 1:5000) antibody at 4°C overnight. After washing, the membrane was incubated with an HRP-conjugated anti-rabbit/mouse IgG (7074S, Cell Signaling Technology, 1:5000) for 1 hr at room temperature. Signals were detected using Clarity™ Western ECL substrate (Bio-Rad, Mississauga, ON, Canada).

### Enzyme-Linked Immunosorbent Assay (ELISA)

Conditioned media and cell lysates were collected 48 hr after transfection or 24 hr after treatment with TGFβ2. The media were centrifuged at 8000 rpm for 5 min to remove cell debris. Cell lysates from the same cell number in each group were extracted with RIPA buffer containing protease and phosphatase inhibitors. The level of IL1β in cell lysates or conditioned media was determined with a human IL1β PicoKine™ ELISA kit (Boster Bio, Pleasanton, CA, USA) and analyzed using a BioTek Synergy H4 hybrid multi-mode plate reader.

### Wound Healing Assay

Cell migration was determined using an IncuCyte^®^ scratch wound healing approach. IncuCyte S3 (Sartorius, Gottingen, Germany) is a live-cell analysis system that can capture the images of cells in culture. At 12 hr post-transfection, 3×10^5^ cells were seeded into a 96-well ImageLock plate (Sartorius) and cultured overnight. When cells reached 100% confluence, the ImageLock plate was placed into the WoundMaker (Sartorius) to create a scratch in each well. Cells were then cultured in a FBS-free medium, and the healing process was imaged every 2 or 3 hr with the IncuCyte S3 system. The relative migration rate at different time points was compared with that of 0 hr using the IncuCyte scratch wound analysis module.

### Tube Formation Assay

The ability of trophoblasts to form endothelial-like networks was assessed using tube formation assay, as described previously (19). Briefly, cells were labeled with a green cell-tracking CMFDA dye (Sigma-Aldrich) and were then seeded into a 96-well plate precoated with Cultrex reduced growth factor base membrane extract (RGF-BME) (Trevigen). IncuCyte S3 was used to capture fluorescent images every 2 hr at 4X. Total network length was quantified by Angiogenesis analyzer, a plugin of ImageJ (41).

### Luciferase Reporter Assay

The 3’UTR fragment (12738~13358 nt) of the human *SMAD2* gene containing the putative binding site of miR-218-5p was amplified by PCR and cloned into the pMIR-REPORT luciferase plasmid vector (Thermo Fisher Scientific) at the SpeI and HindIII sites, downstream of a firefly luciferase gene. The sequences of primers for the cloning are listed in **Table 1**. The insertion of the fragment was confirmed by DNA sequencing. For luciferase reporter assay, cells were seeded into a 24-well plate and reached 70% confluence before transfection. Cells were co-transfected with 800 ng SMAD2 3’UTR reporter plasmid, 10 ng Renilla luciferase vector (pCMV-Renilla, Promega), and 80 nM miR-218-5p mimic or negative control (NC) (Shanghai GenePharma) for 6 hr, using Lipofectamine 2000 reagent. At 42 hr following the transfection, cell lysates were harvested, and the luciferase reporter activity was examined using a dual luciferase assay kit (GeneCopoeia, Rockville, MD, USA). Light emission was measured using a BioTek Synergy H4 hybrid multi-mode plate reader.

TGFβ/SMAD signaling activity was measured with pAR3-Lux (a gift from Dr. Jeff Wrana; Addgene plasmid # 24643) and SBE4-Luc (42) (Addgene plasmid #16495) reporter constructs. Control or mir-218-1-overexpressing cells were seeded into 12-well plates and were co-transfected with 1 µg pAR3-Lux (or SBE4-Luc) reporter and 20 ng Renilla luciferase vector (pRL-TK, Promega) using Lipofectamine 2000. At 24 hr after transfection, cells were treated with recombinant human TGFβ1, TGFβ2, TGFβ3 (10 ng/ml), or Activin A (50 ng/ml) for 30 min. These concentrations were chosen based on results from previous studies (10, 14, 43, 44). Cell lysates were then collected, and the dual luciferase activity was examined as described above.

### Statistical Analysis

All statistical analyses were performed using the GraphPad Prism 8 software. A two-tailed Student’s *t*-test was applied to compare the difference between two groups. One-way ANOVA with Tukey’s multiple comparison tests was used for comparisons among multiple groups. Two-way ANOVA with Tukey’s multiple comparison tests was used in the wound healing assay. Most experiments were performed in triplicate but wound healing and tube formation experiments had n=5 or more. All experiments were repeated at least 2 times. The Shapiro-Wilk test was used to confirm that all data followed normal distribution before the *t*-test or ANOVA analysis. No outliers were identified using the ROUT method integrated with the software. Results were considered significant with a *p*-value less than 0.05.

## Results

### miR-218-5p Induces IL1β Expression and Secretion

**Figure 1:**
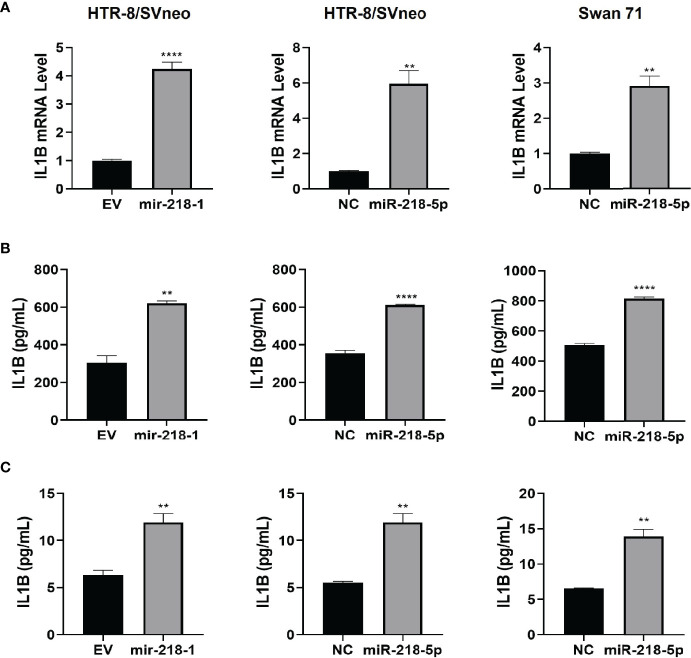
miR-218-5p induces IL1β expression and secretion. Stable transfection of mir-218-1 (left) in HTR-8/SVneo cells, transient transfection of miR-218-5p mimic in HTR-8/SVneo (middle) and Swan 71 (right) cells significantly increased *IL1B* mRNA **(A)** and IL1β protein levels in cell lysates **(B)** and conditioned media **(C)**. Data are shown as mean ± SEM (n=3). **p < 0.01; ****p < 0.0001. EV, empty vector; NC, non-targeting control.

We have previously reported that in HTR-8/SVneo cells, miR-218-5p increased IL1β expression (28). Here, we first verified the upregulation of IL1β production by miR-218-5p. qPCR assay showed that the expression of *IL1B* mRNA was markedly elevated in HTR-8/SVneo cells stably transfected with mir-218-1, and in HTR-8/SVneo and Swan 71 cells transiently transfected with miR-218-5p mimic, compared to that of the control cells (**Figure 1A**). ELISA was also performed to measure IL1β in cell lysates and conditioned media. As shown in **Figures 1B, C**, IL1β protein levels were increased in both lysates and media harvested from cells that had been transfected with mir-218-1 or miR-218-5p. These results suggest that miR-218-5p induces IL1β expression and secretion.

### miR-218-5p Inhibits SMAD Signaling by Targeting SMAD2

**Figure 2:**
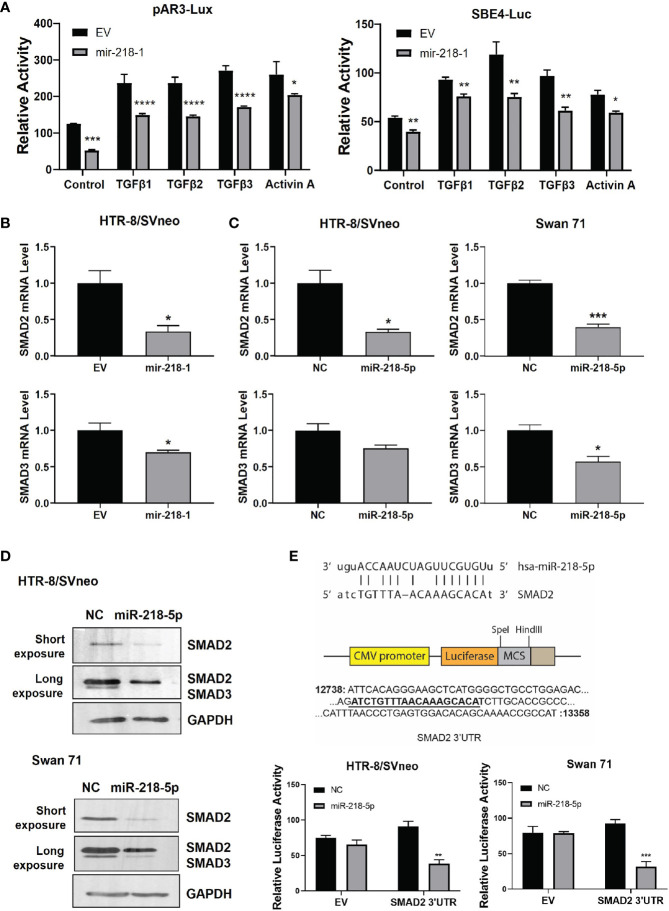
miR-218-5p inhibits SMAD2 by directly binding to its 3’UTR. **(A)** Reporter assay using two SMAD-responsive luciferase reporter vectors, pAR3-Lux and SBE4-Luc. Treatment with TGFβ or Activin A increased SMAD2/3 transcriptional activity but their effects were lower in mir-218-1-overexpressing HTR-8/SVneo cells than in control cells. **(B)** qPCR for *SMAD2* and *SMAD3* mRNA in control and mir-218-1-overexpressing HTR-8/SVneo cells. **(C)** qPCR for *SMAD2* and *SMAD3* mRNA in control and miR-218-5p mimic-treated HTR-8/SVneo and Swan 71 cells. **(D)** Western blotting for SMAD2 and SMAD3 in cells treated with miR-218-5p mimic. Data are representative of three independent experiments. **(E)** miR-218-5p targets SMAD2 3’UTR. A PCR fragment containing a predicted miR-218-5p binding site was cloned into the pMIR-REPORT vector downstream of the luciferase gene. miR-218-5p decreased the luciferase activity of the SMAD2 3’UTR reporter vector. Data are shown as mean ± SEM (n=3). *p < 0.05, **p < 0.01, ***p < 0.001, ****p < 0.0001. EV, empty vector; NC, non-targeting control.

We have reported that miR-218-5p targets TGFB2, leading to reduced SMAD2/3 transcriptional activity (28). To further investigate the regulation of miR-218-5p on the TGFβ/SMAD pathway, we performed a luciferase reporter assay in control and mir-218-1-overexpressing HTR-8/SVneo cells to measure the activity of SMAD2/3 in regulating transcription. Using two SMAD2/3 responsive reporter constructs, pAR3-Lux and SBE4-Luc, we found that mir-218-1 overexpression resulted in a decrease in not only basal, but also TGFβ- and Activin-activated SMAD2/3 transcriptional activity (**Figure 2A**). This result raised the possibility that miR-218-5p may target SMAD2 and/or SMAD3 directly. Therefore, we determined whether miR-218-5p regulates SMAD2 and SMAD3 expression levels. In mir-218-1-overexpressing HTR-8/SVneo cells, *SMAD2*, and to a lesser extent, *SMAD3* mRNA levels were downregulated (**Figure 2B**). Similarly, transient transfection of miR-218-5p mimic decreased SMAD2 and SMAD3 at both mRNA (**Figure 2C**) and protein (**Figure 2D**) levels in HTR-8/SVneo and Swan 71 cells. Notably, in both cell lines, the SMAD2 protein level was much higher than SMAD3 (**Figure 2D**). In addition, miR-218-5p could also upregulate inhibitory SMADs (SMAD6 and SMAD7) to inhibit SMAD signals. SMAD7 is known to block the activation of R-SMADs by TGFβ, activin, and bone morphogenetic proteins (BMPs), while SMAD6 preferentially inhibits BMP-induced SMAD1 activation (45, 46). Therefore, we also tested if miR-218-5p regulates SMAD7. qPCR assay showed that *SMAD7* mRNA was significantly upregulated by miR-218-5p in the two cell lines (**Figure S1**).

Using the bioinformatics tool miRanda (47), we identified a potential miR-218-5p binding site in the 3’UTR of the *SMAD2* gene; however, no miR-218-5p binding sites were predicted in both the coding region and 3’UTR of *SMAD3*. We then generated a luciferase reporter construct by inserting a fragment of SMAD2 3’UTR containing the predicted miR-218-5p binding site into the pMIR-REPORT vector, downstream of the luciferase coding sequence. Reporter assays showed that transfection of miR-218-5p mimic inhibited the luciferase activity in both cell lines (**Figure 2E**). These results suggest that miR-218-5p directly targets the *SMAD2* gene.

### TGFβ2 Suppresses IL1β Expression *via* SMAD2

**Figure 3:**
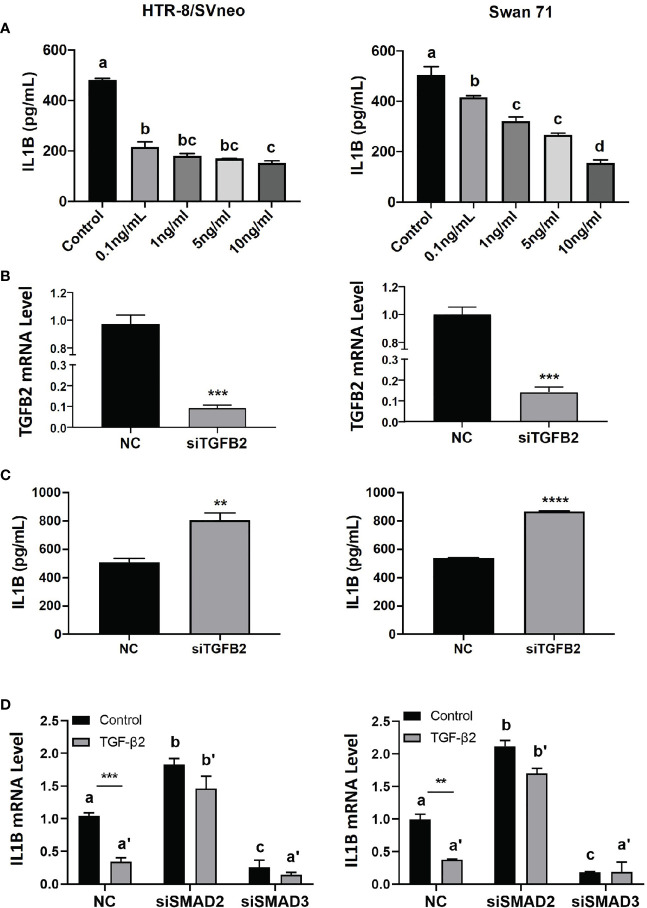
TGFβ2 inhibits IL1β *via* SMAD2 signaling. **(A)** ELISA for IL1β in cell lysates harvested from HTR-8/SVneo and Swan 71 cells treated with recombinant TGFβ2 at various concentrations for 24 hr. **(B)** qPCR for *TGFB2* mRNA in cells transfected with siTGFB2. **(C)** ELISA for IL1β in cell lysates harvested from cells treated with siTGFB2. **(D)** qPCR for *IL1B* mRNA in control, siSMAD2, and siSMAD3-treated cells, in the presence of recombinant TGFβ2 (1 ng/ml). Data are shown as mean ± SEM (n=3). **p < 0.01, ***p < 0.001, ****p < 0.0001. For **(A, D)**, different letters above bars denote statistical significance.

To further investigate the effect of TGFβ2/SMAD2 on IL1β expression, we treated HTR-8/SVneo and Swan 71 cells with recombinant TGFβ2 or transiently transfected with siRNA targeting the *TGFB2* gene (siTGFB2). ELISA showed that TGFβ2 dose-dependently reduced IL1β protein level in both cell lines (**Figure 3A**). Conversely, siTGFB2, which strongly decreased *TGFB2* mRNA (**Figure 3B**), significantly increased IL1β protein expression (**Figure 3C**). We also transfected siRNA targeting SMAD2 or SMAD3 into HTR-8/SVneo and Swan 71 cells and then treated cells with TGFβ2. Consistent with our previous report (19), knockdown of SMAD2 upregulated, while knockdown of SMAD3 downregulated *IL1B* mRNA level (**Figure 3D**). Interestingly, SMAD2 siRNA also completely reversed the inhibitory effect of TGFβ2 on *IL1B* expression. However, TGFβ2 still strongly inhibited *IL1B* mRNA in cells transfected with siSMAD3 (**Figure 3D**). These results suggest that SMAD2, but not SMAD3, is required for TGFβ2 to inhibit IL1β expression.

### SMAD2 Blocks miR-218-5p-Induced *IL1B* mRNA

**Figure 4:**
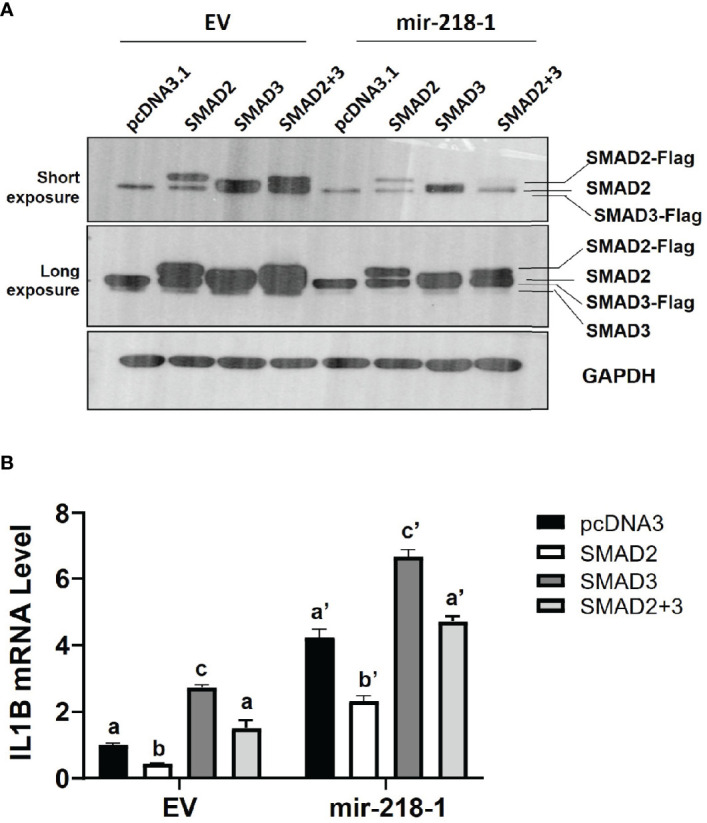
SMAD2 blocks miR-218-5p-induced *IL1B* mRNA. **(A)** Western blotting for SMAD2 and SMAD3 in control and mir-218-1-overexpressing HTR-8/SVneo cells transiently transfected with Flag-tagged SMAD2 and/or SMAD3 expression constructs. Note that mir-218-1 overexpression reduced both endogenous and exogenous SMAD2 and SMAD3 protein levels. Data are representative of three independent experiments. **(B)** qPCR for *IL1B* mRNA in control and mir-218-1-overexpressing HTR-8/SVneo cells transfected with SMAD2 and/or SMAD3 vectors, showing that SMAD2 and SMAD3 exerted opposing effects on *IL1B* mRNA. Data are shown as mean ± SEM (n=3). Different letters above bars denote statistical significance.

We next investigated the role of SMAD2 and SMAD3 in miR-218-5p-mediated IL1β upregulation. mir-218-1-overexpressing or control cells were transfected with a Flag-tagged SMAD2 and/or SMAD3 expression plasmids. Western blotting analysis confirmed the expression of exogenous SMAD2 and SMAD3 after transfection (**Figure 4A**). As expected, endogenous SMAD2 and SMAD3 levels were lower in mir-218-1 stable cells than in control cells. Surprisingly, exogenous SMAD2 and SMAD3 levels were also reduced in mir-218-1-overexpressing cells (**Figure 4A**). As shown in **Figure 4B**, overexpression of SMAD2 reduced basal and mir-218-1-induced *IL1B* mRNA; however, SMAD3 overexpression increased *IL1B* in both control and mir-218-1-overexpressing cells. In addition, co-transfection of SMAD2 and SMAD3 constructs showed no apparent difference in *IL1B* mRNA when compared to that of cells transfected with empty vectors. These results suggest that SMAD2 blocks, while SMAD3 enhances, miR-218-5p-induced *IL1B* expression and that SMAD2 and SMAD3 may have antagonistic effects on regulating IL1β.

### SMAD2 Inhibits miR-218-5p-Induced Acquisition of an enEVT-Like Phenotype

**Figure 5:**
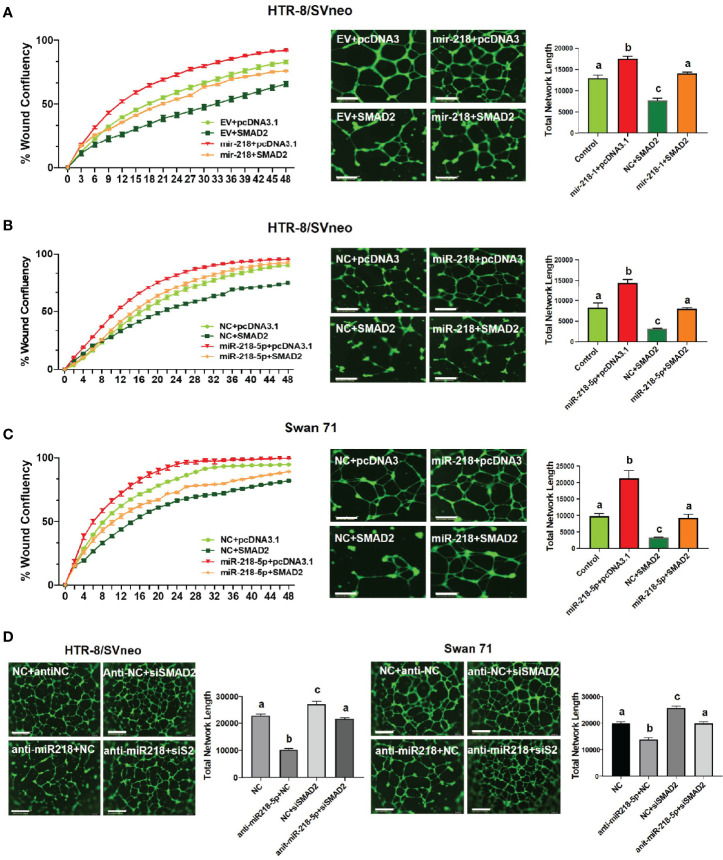
SMAD2 inhibits miR-218-5p-induced cell migration and formation of endothelial-like networks. **(A)** Wound healing (left, n=6) and tube formation (right, n=5-6) assays in control or mir-218-1-overexpressing cells, transfected with SMAD2-expressing construct or its control pcDNA3.1 vector. **(B, C)** Wound healing (left, n=6) and tube formation (right, n=5-6) assays in HTR-8/SVneo and Swan71 cells, co-transfected with miR-218-5p mimic, SMAD2 construct, or their non-targeting control (NC) or empty vector, pcDNA3.1. Note that SMAD2 overexpression reversed the stimulatory effects of miR-218-5p on wound closure and the formation of endothelial-like networks. **(D)** Silencing of SMAD2 partially abolished the inhibitory effect of anti-miR-218-5p on endothelial-like network formation (n=6). Data are shown as mean ± SEM; scale bar = 800 µm. For wound healing assay in **(A–C)**, the differences among all groups are significant (*p* < 0.05) starting at 10 hr.

We have previously demonstrated that miR-218-5p induces (28), while SMAD2 suppresses (19), the acquisition of an enEVT-like phenotype in trophoblasts. To investigate if miR-218-5p induces enEVT differentiation by targeting SMAD2, we performed functional rescue experiments. The acquisition of an enEVT phenotype was assessed by wound healing and tube formation assays. miR-218-5p accelerated cell migration in the wound healing assay (**Figures 5A–C**, left) and enhanced the formation of endothelial-like networks (**Figures 5A–C**, right). However, SMAD2 overexpression repressed both baseline and miR-218-5p-promoted migratory and network forming capacities of trophoblasts (**Figures 5A** **–C)**. Conversely, silencing of SMAD2 abolished anti-miR-218-5p-reduced endothelial-like network formation in the two cell lines (**Figure 5D**). These results suggest that miR-218-5p induces enEVT differentiation, in part, by downregulating SMAD2.

### IL1β Induces the Acquisition of an enEVT-Like Phenotype

**Figure 6:**
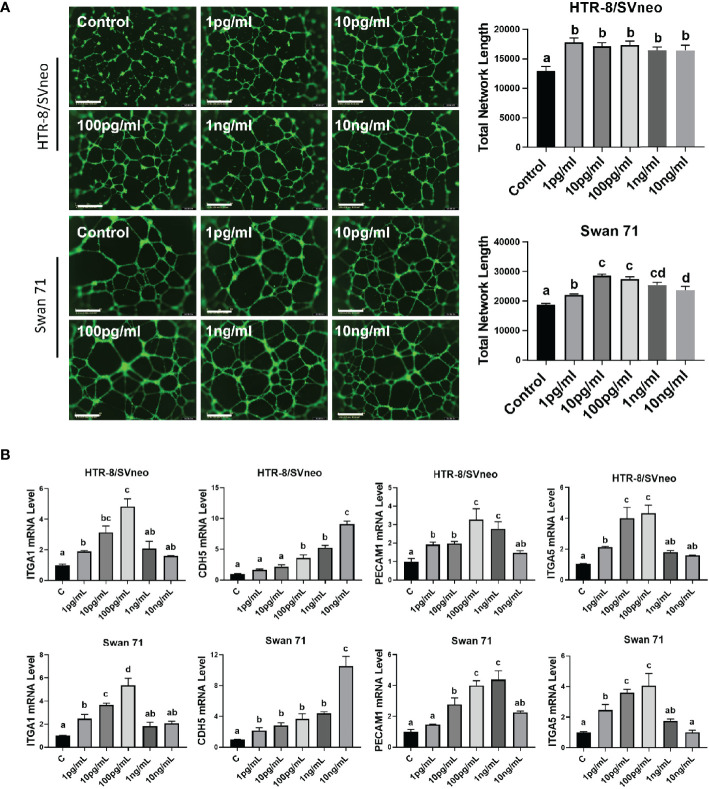
IL1β promotes the acquisition of an enEVT-like phenotype. **(A)** Tube formation assay showing that IL1β enhanced the ability of HTR-8/SVneo and Swan 71 cells to form endothelial-like networks (n=6); scale bar = 800 µm. **(B)** qPCR for the expression of enEVT markers in cells treated with IL1β (n=3). Note that IL1β at low concentrations increased *ITGA1*, *ITGA5*, *CDH5*, and *PECAM1* mRNA in a dose-dependent manner. Data are shown as mean ± SEM. Different letters above bars denote statistical significance.

IL1β can enhance the invasive ability of primary EVTs (32); however, whether it is involved in enEVT differentiation is unknown. Therefore, we explored the role of IL1β in the induction of an enEVT-like phenotype. We used recombinant IL1β at concentrations of 1 pg/ml–10 ng/ml in functional assays, a dosage range commonly used in previous studies (32, 48, 49). We found that IL1β enhanced the ability of trophoblasts to form endothelial-like network structures starting from 1 pg/ml (**Figure 6A**). IL1β also elevated the mRNA levels of enEVT markers, including integrin subunit α1 (ITGA1), ITGA5, cadherin 5 (CDH5, also known as vascular endothelial cadherin, VE-Cadherin), and platelet endothelial cell adhesion molecule 1 (PECAM1) in the two cell lines (**Figure 6B**) in a dosage range of 1 pg/ml–100 pg/ml. However, IL1β at higher concentrations (1 ng/ml–10 ng/ml) had little effect in the induction of these marker genes (except for *CDH5*). Taken together, these results suggest that IL1β may promote enEVT differentiation.

### miR-218-5p Is Negatively Regulated by IL1β

**Figure 7:**
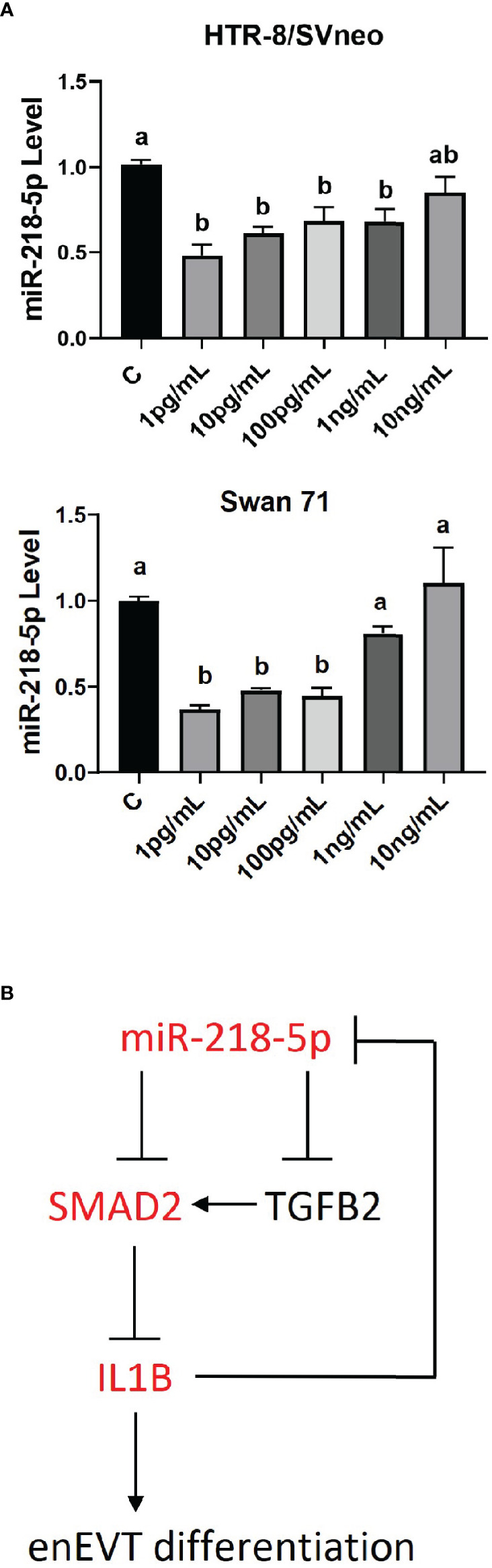
Regulation of miR-218-5p by IL1β. **(A)** HTR-8/SVneo and Swan 71 cells were treated with different concentrations of IL1β for 24 hr. qPCR revealed that lower doses of IL1β reduced miR-218-5p level. Data are shown as mean ± SEM (n=3). Different letters above bars denote statistical significance. **(B)** A schematic outlining the interactions among miR-218-5p, IL1β, and the TGFβ/SMAD2 pathway and their potential roles in enEVT differentiation.

microRNAs are involved in regulatory networks of cytokines and growth factors (50–52). We therefore determined whether IL1β could, in turn, affect miR-218-5p expression. As shown in **Figure 7A**, IL1β at lower doses (1 pg/ml–100 pg/ml) inhibited miR-218-5p in HTR-8/SVneo and Swan 71 cells. However, higher doses of IL1β didn’t alter miR-218-5p expression. These results suggest that lower concentrations of IL1β exert negative feedback on miR-218-5p (**Figure 7B**).

## Discussion

In this study, we reported that miR-218-5p induces IL1β to promote the acquisition of an enEVT-like phenotype. IL1β is increased by miR-218-5p but decreased by TGFβ2/SMAD2. Mechanistically, miR-218-5p induces IL1β through the suppression of the SMAD2-mediated TGFβ signaling. On the other hand, IL1β also exerts a negative feedback modulation on miR-218-5p expression (**Figure 7B**). These findings suggest an interactive network of miR-218-5p, IL1β, and the TGFβ/SMAD2 pathway, which regulates enEVT differentiation.

Although several studies have shown that IL1β increases trophoblast migration and viability (31–33), its role in EVT differentiation is unknown. Recently, we reported that miR-218-5p induces enEVT differentiation and increases IL1β expression (28). On the other hand, miR-210-3p inhibits the acquisition of an enEVT phenotype and also reduces IL1β expression (53). In this study, we showed that IL1β increased the expression of several enEVT differentiation-associated markers, such as ITGA1, ITGA5, CDH5, and PECAM1. IL1β also accelerated cell migration and the formation of endothelium-like networks. These findings suggest that IL1β functions as a positive regulator of enEVT differentiation. IL1β has been reported to be released from decidual uterine NK cells, stromal cells, and macrophages (54, 55). Previous reports (19, 56, 57) and this study also revealed that both HTR-8/SVneo and Swan 71 cells expressed and secreted IL1β, supporting paracrine/autocrine effects of IL1β on the acquisition of an enEVT phenotype. Interestingly, we found that except for CDH5, IL1β significantly stimulated the expression of enEVT markers at 1 pg/ml and the maximal effect was observed at a dose of 100 pg/ml or 1 ng/ml, while higher doses had lower or no effects on the enEVT marker gene expression. The effective doses of IL1β in inducing the formation of endothelial-like networks and expression of enEVT markers are within the range of what we detected in the conditioned media from HTR-8/SVneo and Swan 71 cells. Thus, IL1β likely promotes enEVT differentiation only under physiological conditions, yet a high-level IL1β may have adverse or even detrimental outcomes. Although inflammation is a critical component during normal pregnancies, maintaining a physiological balance of pro- and anti-inflammatory cytokines is essential for a successful pregnancy. As a major pro-inflammatory cytokine, high levels of IL1β may directly participate in the extensive inflammatory response that is correlated with pregnancy complications including PE (58, 59). Further, IL1β is known to act as a potential mediator of endothelial dysfunction by inducing structural and functional alterations in endothelial cells (59–61), which is a hallmark of the maternal syndrome in PE.

We have previously reported that miR-218-5p expression is lower in PE placentas than in healthy controls and that this miRNA enhances enEVT differentiation and spiral artery remodeling by targeting TGFβ2 ligand (28). In this study, overexpression of mir-218-1 or treatment with miR-218-5p mimic decreased SMAD2 at both mRNA and protein levels in HTR-8/SVneo and Swan 71 trophoblasts. The reporter assay verified the direct binding of miR-218-5p to the 3’UTR of the *SMAD2* gene. Interestingly, in mir-218-1-overexpressing cells, both endogenous and transiently overexpressed exogenous SMAD2 protein levels were lower than those of the control cells. Since the SMAD2 expression construct does not contain a 3’UTR, this decrease cannot be explained by the binding of miR-218-5p to the SMAD2 3’ UTR. Therefore, it is likely that miR-218-5p also regulates the stability of SMAD2. Furthermore, we found that SMAD7, which can inhibit SMAD2/3 activation by the TGFβ family (45, 46), was significantly upregulated by miR-218-5p. Although the role of miR-218-5p in regulating SMAD7 expression and SMAD2 protein stability requires further investigation, these findings suggest that miR-218-5p inhibits SMAD2 activity *via* multiple direct and indirect actions. To validate that miR-218-5p modulates cellular behaviors of trophoblasts through inhibition of SMAD2, we performed a series of functional assays in the two cell lines. We found that SMAD2 overexpression reduced the migration and the ability to form endothelium-like networks in both control and miR-218-5p-treated trophoblasts. On the other hand, SMAD2 knockdown increased the formation of the endothelial networks in control and anti-miR-218-5p-treated cells. These data suggest that miR-218-5p stimulates the acquisition of an enEVT-like phenotype by targeting both TGFβ2 and SMAD2, thus leading to impaired TGFβ/SMAD2 signaling.

In this study, we found that both HTR-8/SVneo and Swan 71 cells treated with miR-218-5p mimic or transfected with *mir-218-1* transgene displayed increased expression/secretion of IL1β. In contrast, TGFβ2 treatment reduced IL1β production, whereas siTGFB2 induced IL1β protein level. In a previous study, we showed that SMAD2 knockdown in HTR-8/SVneo cells stimulates the expression of several genes involved in trophoblast differentiation and function, such as *MMP1*, *CDH5*, *IL8*, and *IL1B* (19). Here, we confirmed that silencing of SMAD2 upregulated *IL1B* mRNA in two trophoblast cell lines. Further, we showed that SMAD2 knockdown attenuated the inhibitory effect of TGFβ2, while SMAD2 overexpression abolished the stimulatory effect of miR-218-5p, on *IL1B* expression. These findings, together with the inhibition of TGFβ2 and SMAD2 by miR-218-5p, indicate that miR-218-5p induces *IL1B* by downregulating the TGFβ2/SMAD2 pathway.

Several studies have reported opposing actions of TGFβ and IL1β, particularly in immune and hematopoietic systems (62–64). For example, TGFβ1 inhibits IL1β-induced IL6 and IL17 in monocytes and CD4+ T cells, respectively (65, 66). In mouse calvarial osteoblasts, TGFβ abolishes the induction of cyclooxygenase 2 by IL1β (67). TGFβ and IL1β also antagonistically modulate apoptosis of corneal myoblasts (68). In trophoblasts, TGFβ and IL1β have inhibitory and stimulatory effects, respectively, on cell invasion (32, 69, 70). In this study, we showed that TGFβ and IL1β displayed opposite effects not only on cell migration, but also on the expression of enEVT markers and the formation of endothelial-like networks. Additionally, we demonstrated that TGFβ, signaling *via* SMAD2, inhibited IL1β expression. The mechanism by which TGFβ/SMAD2 represses IL1β is not known and remains to be investigated in the future.

Consistent with our recent report (19), we observed that SMAD2 downregulated, while SMAD3 upregulated IL1β in trophoblasts. Although the two SMAD molecules share 92% amino acid sequence identity (71), they are not functionally equivalent and may play non-overlapping or even disparate roles in physiological and pathological conditions. For instance, SMAD3 differs from SMAD2 in static subcellular localization, the ability and sensitivity to transmit TGFβ signal, and early lineage specification (72). In pancreatic cancer cells, Rac1 represses the TGFβ1-mediated growth inhibition by suppressing SMAD2 but activating SMAD3 (73). Recently, we reported that SMAD2 blocks the acquisition of an enEVT-like phenotype but SMAD3 shows an opposite effect (19). Findings from the present study further support the differential functions of SMAD2 and SMAD3 in this process.

Although SMAD3 upregulates IL1β, a function similar to that of miR-218-5p, we found that SMAD3 mRNA and protein levels were also reduced by miR-218-5p. Unlike the *SMAD2* gene that harbors a binding site of miR-218-5p in its 3’UTR, SMAD3 appears not a direct target as no predicted miR-218-5p binding sites were identified in SMAD3 3’UTR and coding region. Hence, miR-218-5p likely downregulates SMAD3 using some indirect unknown mechanisms, possibly *via* its other target genes. Using an antibody that detects both SMAD2 and SMAD3, we found that the endogenous SMAD2 protein level was much higher than SMAD3 in both trophoblast cell lines, indicating a differential abundance of the two SMAD proteins. It has been shown that the ratio of SMAD2 to SMAD3 is cell type-dependent and may be a determinant for the relative sensitivity of SMAD2 or SMAD3 to TGFβ signals (74). For example, a decreased SMAD2/SMAD3 ratio enhances the SMAD3-dependent pathway in response to TGF-β (74). In this study, although SMAD3 upregulates IL1β, its expression is much lower than SMAD2. It is possible that the high SMAD2/SMAD3 ratio ensures that SMAD2, rather than SMAD3, predominantly mediates the innate TGFβ signals.

Interestingly, while IL1β is stimulated by miR-218-5p, treatment with IL1β at lower dosages also reduced miR-218-5p expression in HTR-8/SVneo and Swan 71 cells, with the highest concentration tested (10 ng/ml) exhibiting no effect. These findings suggest that IL1β at physiological concentrations exerts negative feedback on miR-218-5p expression to limit its induction of IL1β. This self-regulatory property may be helpful to maintain IL1β at a moderate level to properly modulate trophoblast differentiation. On the other hand, an imbalanced IL1β overproduction, primarily induced under pathological conditions (e.g., infection), may lose its ability to inhibit miR-218-5p and is associated with harmful effects (such as extensive inflammation and endothelial dysfunction) that are implicated in the pathogenesis of PE.

In summary, we have demonstrated that miR-218-5p induces enEVT differentiation in part by inhibiting the TGFβ2/SMAD2 pathway, leading to enhanced IL1β expression and secretion. We also identified IL1β-mediated negative feedback on miR-218-5p expression. These findings highlight a novel interactive miR-218-5p/TGFβ/SMAD2/IL1β signaling nexus that plays an important role in the acquisition of an enEVT phenotype. To date, although preemptive administration with aspirin, calcium, or metformin can effectively prevent PE, there are no curative treatments for this progressive disorder, and once diagnosed, the only option is delivery (3). As such, understanding the signaling mechanism that underscores enEVT differentiation can facilitate the development of novel therapeutic strategies for the clinical intervention of PE.

## Data Availability Statement

The raw data supporting the conclusions of this article will be made available by the authors, without undue reservation.

## Author Contributions

YS and CP designed the study. YS, YC, JB, LF, and HM performed experiments and collected data. YS, YC, and CP analyzed the data. YS, YC, and CP wrote the manuscript. All authors have read, edited, and approved this submission.

## Funding

This study was supported by funding from the Canadian Institutes of Health Research (CIHR, PJT-153146), Canadian Foundation for Innovation/Ontario Research Fund (Project # 35611), and York Research Chair Program to CP.

## Conflict of Interest

The authors declare that the research was conducted in the absence of any commercial or financial relationships that could be construed as a potential conflict of interest.

## Publisher’s Note

All claims expressed in this article are solely those of the authors and do not necessarily represent those of their affiliated organizations, or those of the publisher, the editors and the reviewers. Any product that may be evaluated in this article, or claim that may be made by its manufacturer, is not guaranteed or endorsed by the publisher.
