# Supplementary material for: miR-218-5p Induces Interleukin-1β and Endovascular Trophoblast Differentiation by Targeting the Transforming Growth Factor β-SMAD2 Pathway
Source: Front Endocrinol (Lausanne). 2022 Mar 1;13:842587. doi: 10.3389/fendo.2022.842587 (PMC8920978; doi:10.3389/fendo.2022.842587)
Supplement: Supplementary file 1 [file DataSheet_1.pdf]

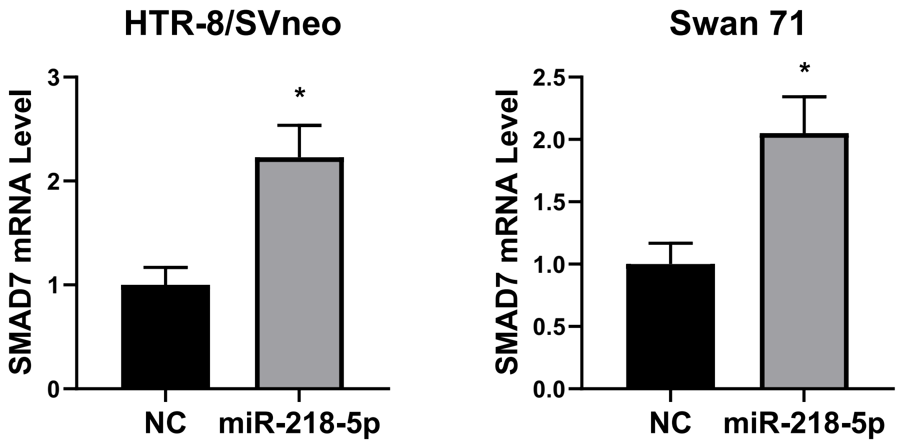

Supplemental Figure 1. SMAD7 is upregulated by miR-218-5p. HTR-8/SVneo and Swan 71 cells were transiently transfected with miR-218-5p mimic or negative control oligo (NC). Real-time PCR revealed that miR-218-5p increased *SMAD7* mRNA. Data present mean ± SEM (n=3). * p<0.05.
